# Opipramolium fumarate

**DOI:** 10.1107/S160053681103159X

**Published:** 2011-08-11

**Authors:** M. S. Siddegowda, Jerry P. Jasinski, James A. Golen, H. S. Yathirajan, M. T. Swamy

**Affiliations:** a Department of Studies in Chemistry, University of Mysore, Manasagangotri, Mysore 570 006, India; b Department of Chemistry, Keene State College, 229 Main Street, Keene, NH 03435-2001, USA; c Department of Chemistry, Sambhram Institute of Technology, Bangalore, 560 097, India

## Abstract

In the crystal structure of the title salt {systematic name: 4-[3-(5*H*-dibenz[*b*,*f*]azepin-5-yl)prop­yl]-1-(2-hy­droxy­eth­yl)piperazin-1-ium (2*Z*)-3-carb­oxy­prop-2-enoate}, C_23_H_30_N_3_O^+^·C_4_H_3_O_4_^−^, the piperazine group in the opipramol cation is protonated at only one of the N atoms. In the cation, the dihedral angle between the two benzene rings is 53.5 (6)°. An extensive array of inter­molecular O—H⋯O, O—H⋯N and N—H⋯O hydrogen bonds and weak inter­molecular N—H⋯O, C—H⋯O and C—H⋯π inter­actions dominate the crystal packing.

## Related literature

For the use of opipramol in the treatment of anxiety disorders, see: Moller *et al.* (2001 ▶). For related structures, see: Fun *et al.* (2011 ▶); Jasinski *et al.* (2010 ▶). For standard bond lengths, see Allen *et al.* (1987 ▶).
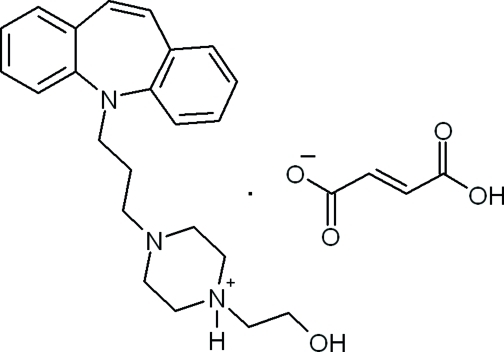


## Experimental

### 

#### Crystal data

C_23_H_30_N_3_O^+^·C_4_H_3_O_4_^−^
*M* *_r_* = 479.56
Monoclinic, 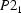

*a* = 8.9116 (3) Å
*b* = 6.7167 (3) Å
*c* = 20.6377 (8) Å
β = 98.685 (3)°
*V* = 1221.14 (8) Å^3^
*Z* = 2
Mo *K*α radiation
μ = 0.09 mm^−1^
*T* = 173 K
0.25 × 0.22 × 0.12 mm

#### Data collection

Oxford Diffraction Xcalibur Eos Gemini diffractometer
Absorption correction: multi-scan (*CrysAlis RED*; Oxford Diffraction, 2010 ▶) *T*_min_ = 0.978, *T*_max_ = 0.989
8285 measured reflections
3393 independent reflections
3116 reflections with *I* > 2σ(*I*)
*R*_int_ = 0.029

#### Refinement

*R*[*F* ^2^ > 2σ(*F* ^2^)] = 0.038
*wR*(*F* ^2^) = 0.097
*S* = 1.03
3393 reflections
325 parameters
4 restraints
H atoms treated by a mixture of independent and constrained refinement
Δρ_max_ = 0.26 e Å^−3^
Δρ_min_ = −0.19 e Å^−3^

### 

Data collection: *CrysAlis PRO* (Oxford Diffraction, 2010 ▶); cell refinement: *CrysAlis PRO*; data reduction: *CrysAlis RED* (Oxford Diffraction, 2010 ▶); program(s) used to solve structure: *SHELXS97* (Sheldrick, 2008 ▶); program(s) used to refine structure: *SHELXL97* (Sheldrick, 2008 ▶); molecular graphics: *SHELXTL* (Sheldrick, 2008 ▶); software used to prepare material for publication: *SHELXTL*.

## Supplementary Material

Crystal structure: contains datablock(s) global, I. DOI: 10.1107/S160053681103159X/bt5601sup1.cif

Structure factors: contains datablock(s) I. DOI: 10.1107/S160053681103159X/bt5601Isup2.hkl

Supplementary material file. DOI: 10.1107/S160053681103159X/bt5601Isup3.cml

Additional supplementary materials:  crystallographic information; 3D view; checkCIF report

## Figures and Tables

**Table 1:** Hydrogen-bond geometry (Å, °) *Cg*2 and *Cg*3 are the centroids of the C1–C6 and C9–C14 rings, respectively.

*D*—H⋯*A*	*D*—H	H⋯*A*	*D*⋯*A*	*D*—H⋯*A*
O1—H1*O*⋯O4^i^	0.85 (2)	1.83 (2)	2.674 (2)	171 (3)
O2—H2*O*⋯N2^ii^	0.87 (2)	1.79 (2)	2.649 (2)	176 (3)
N3—H3*N*⋯O5	0.90 (2)	1.72 (2)	2.616 (2)	179 (2)
N3—H3*N*⋯O4	0.90 (2)	2.59 (2)	3.167 (2)	123 (2)
C12—H12*A*⋯O3^iii^	0.95	2.49	3.399 (3)	159
C19—H19*A*⋯O2^iv^	0.99	2.57	3.551 (2)	170
C19—H19*B*⋯O1^v^	0.99	2.47	3.365 (2)	151
C21—H21*B*⋯O5^vi^	0.99	2.43	3.415 (2)	172
C22—H22*B*⋯O1^v^	0.99	2.57	3.447 (2)	148
C2—H2*A*⋯*Cg*2^vii^	0.95	2.95	3.684 (2)	135
C5—H5*A*⋯*Cg*3^viii^	0.95	2.79	3.655 (2)	152

Symmetry codes: (i) 
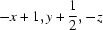
; (ii) 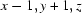; (iii) 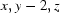; (iv) 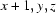; (v) 
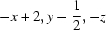
; (vi) 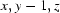; (vii) 
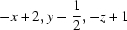
; (viii) 
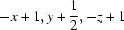
.

**Fig. 1.**
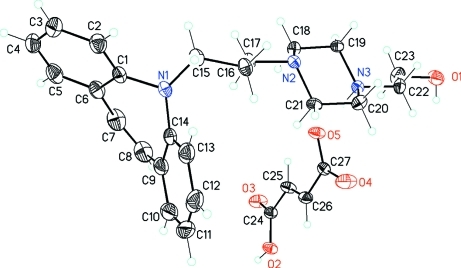
Molecular structure of the title compound showing the atom labeling scheme and 50% probability displacement ellipsoids.

**Fig. 2.**
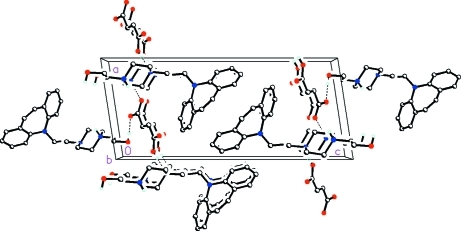
Packing diagram of the title compound viewed down the b axis. Dashed lines indicate O—H···O, O—H···N and N—H···O hydrogen bonds. The hydrogen atoms not involved in H-bonding have been deleted for clarity.

## supplementary crystallographic information

### Comment

Opipramol [systematic IUPAC name: 4-[3-(5*H*-dibenz[b,f] azepin-5-yl)propyl]-1-piperazinethanol] is an antidepressant and anxiolytic typically used in the treatment of generalized anxiety disorder (Moller *et al.*, 2001). Opipramol is a tricyclic compound with no reuptake-inhibiting properties. However, it has pronounced D2-, 5-HT2-, and H1-blocking potential and high affinity to sigma receptors (sigma-1 and sigma-2). The crystal structure studies of opipramol dipicrate (Jasinski *et al.*, 2010) and opipramol (Fun *et al.*, 2011) have been reported. In view of the importance of opipramol, the paper reports the crystal structure of the title compound, (I).

In Opipramolium fumarate, C_23_H_30_N_3_O^+^, C_4_H_3_O_4_^-^,   the piperazine group in the opipramol cation is protonated at only one of the N atoms (Fig. 1). The 6-membered piperazine group (N2/C18/C19/N3/C20/C21) adopts a slightly distorted chair conformation with puckering parameters Q, θ and φ of 0.5894 (18) Å, 2.00 (17)°, and 14 (6)°, respectively. For an ideal chair θ has a value of 0 or 180°. In the cation the dihedral angle between the two benzene rings is 53.5 (6)°. Bond distances and angles are in normal ranges (Allen *et al.*, 1987). An extensive array of O—H···O, O—H···N and N—H···O hydrogen bonds and weak N—H···O, C—H···O, C—H···*Cg*π-ring intermolecular interactions (Table 1), dominate crystal packing in the unit cell (Fig. 2).

### Experimental

Opipramol base (2.0 g, 0.0055 mol) was dissolved in 10 ml of DMSO and fumaric acid (1.276 g, 0.011 mol) was added. The solution was stirred in a beaker at 348 K for 15 minutes. The mixture was kept aside for two days at room temperature. Crystals of the product formed were used as such for *x*-ray work (m. p.: 432–434 K).

### Refinement

The N–H and O–H atoms were located by a difference Fourier map and refined isotropically with *DFIX* = 0.87Å and 0.80Å, respectively. All of the remaining H atoms were placed in their calculated positions and then refined using the riding model with C—H lengths of 0.95Å (CH) or 0.99Å (CH_2_). The isotropic displacement parameters for these atoms were set to 1.19 to 1.21 (CH), or 1.18 to 1.22 (CH_2_) times *U*_eq_ of the parent atom. In the absence of anomalous scatterers, Friedel pairs have been merged.

### Crystal data

**Table:**

C_23_H_30_N_3_O^+^·C_4_H_3_O_4_^−^	*Z* = 2
*M**_r_* = 479.56	*F*(000) = 512
Monoclinic, *P*2_1_	*D*_x_ = 1.304 Mg m^−^^3^
Hall symbol: P 2yb	Mo *K*α radiation, λ = 0.71073 Å
*a* = 8.9116 (3) Å	µ = 0.09 mm^−^^1^
*b* = 6.7167 (3) Å	*T* = 173 K
*c* = 20.6377 (8) Å	Block, colorless
β = 98.685 (3)°	0.25 × 0.22 × 0.12 mm
*V* = 1221.14 (8) Å^3^	

### Data collection

**Table:**

Oxford Diffraction Xcalibur Eos Gemini diffractometer	3393 independent reflections
Radiation source: Enhance (Mo) X-ray Source	3116 reflections with *I* > 2σ(*I*)
graphite	*R*_int_ = 0.029
Detector resolution: 16.1500 pixels mm^-1^	θ_max_ = 28.7°, θ_min_ = 3.3°
ω scans	*h* = −5→12
Absorption correction: multi-scan (*CrysAlis RED*; Oxford Diffraction, 2010)	*k* = −8→9
*T*_min_ = 0.978, *T*_max_ = 0.989	*l* = −27→26
8285 measured reflections	

### Refinement

**Table:**

Refinement on *F*^2^	Primary atom site location: structure-invariant direct methods
Least-squares matrix: full	Secondary atom site location: difference Fourier map
*R*[*F*^2^ > 2σ(*F*^2^)] = 0.038	Hydrogen site location: inferred from neighbouring sites
*wR*(*F*^2^) = 0.097	H atoms treated by a mixture of independent and constrained refinement
*S* = 1.03	*w* = 1/[σ^2^(*F*_o_^2^) + (0.0537*P*)^2^ + 0.1565*P*] where *P* = (*F*_o_^2^ + 2*F*_c_^2^)/3
3393 reflections	(Δ/σ)_max_ = 0.010
325 parameters	Δρ_max_ = 0.26 e Å^−^^3^
4 restraints	Δρ_min_ = −0.19 e Å^−^^3^

### Special details

**Table:**

Geometry. All e.s.d.'s (except the e.s.d. in the dihedral angle between two l.s. planes) are estimated using the full covariance matrix. The cell e.s.d.'s are taken into account individually in the estimation of e.s.d.'s in distances, angles and torsion angles; correlations between e.s.d.'s in cell parameters are only used when they are defined by crystal symmetry. An approximate (isotropic) treatment of cell e.s.d.'s is used for estimating e.s.d.'s involving l.s. planes.
Refinement. Refinement of *F*^2^ against ALL reflections. The weighted *R*-factor *wR* and goodness of fit *S* are based on *F*^2^, conventional *R*-factors *R* are based on *F*, with *F* set to zero for negative *F*^2^. The threshold expression of *F*^2^ > σ(*F*^2^) is used only for calculating *R*-factors(gt) *etc*. and is not relevant to the choice of reflections for refinement. *R*-factors based on *F*^2^ are statistically about twice as large as those based on *F*, and *R*- factors based on ALL data will be even larger.

### Fractional atomic coordinates and isotropic or equivalent isotropic displacement parameters (Å^2^)

**Table:**

	*x*	*y*	*z*	*U*_iso_*/*U*_eq_	
O1	0.84102 (17)	0.6719 (3)	−0.03728 (7)	0.0331 (4)	
H1O	0.753 (2)	0.717 (5)	−0.0516 (12)	0.040*	
O2	0.07743 (14)	0.8233 (2)	0.15863 (7)	0.0229 (3)	
H2O	0.011 (2)	0.898 (4)	0.1734 (11)	0.027*	
O3	0.23640 (16)	1.0656 (2)	0.19905 (8)	0.0322 (4)	
O4	0.42998 (17)	0.3435 (3)	0.07200 (9)	0.0411 (4)	
O5	0.58743 (14)	0.5423 (2)	0.13510 (7)	0.0254 (3)	
N1	0.73872 (17)	−0.2719 (3)	0.37655 (7)	0.0204 (3)	
N2	0.86582 (16)	0.0375 (2)	0.20359 (7)	0.0156 (3)	
N3	0.78846 (17)	0.3093 (2)	0.09390 (7)	0.0161 (3)	
H3N	0.719 (2)	0.388 (3)	0.1083 (10)	0.019*	
C1	0.7716 (2)	−0.2668 (3)	0.44636 (9)	0.0218 (4)	
C2	0.8727 (2)	−0.3992 (4)	0.48113 (10)	0.0290 (5)	
H2A	0.9160	−0.5027	0.4586	0.035*	
C3	0.9113 (3)	−0.3816 (4)	0.54882 (11)	0.0352 (5)	
H3A	0.9824	−0.4711	0.5722	0.042*	
C4	0.8465 (2)	−0.2347 (4)	0.58196 (10)	0.0346 (5)	
H4A	0.8719	−0.2239	0.6282	0.042*	
C5	0.7452 (3)	−0.1040 (4)	0.54813 (10)	0.0332 (5)	
H5A	0.7014	−0.0029	0.5715	0.040*	
C6	0.7047 (2)	−0.1158 (3)	0.48002 (10)	0.0255 (4)	
C7	0.5967 (3)	0.0269 (4)	0.44633 (11)	0.0361 (5)	
H7A	0.5990	0.1571	0.4645	0.043*	
C8	0.4953 (3)	−0.0045 (4)	0.39307 (12)	0.0370 (6)	
H8A	0.4339	0.1056	0.3769	0.044*	
C9	0.4691 (2)	−0.1915 (4)	0.35700 (10)	0.0294 (5)	
C10	0.3209 (2)	−0.2408 (5)	0.32774 (11)	0.0412 (7)	
H10A	0.2406	−0.1498	0.3306	0.049*	
C11	0.2899 (3)	−0.4178 (5)	0.29508 (12)	0.0454 (7)	
H11A	0.1891	−0.4472	0.2753	0.054*	
C12	0.4042 (3)	−0.5525 (5)	0.29095 (11)	0.0424 (6)	
H12A	0.3822	−0.6756	0.2689	0.051*	
C13	0.5525 (3)	−0.5081 (4)	0.31923 (10)	0.0314 (5)	
H13A	0.6313	−0.6016	0.3166	0.038*	
C14	0.5858 (2)	−0.3275 (3)	0.35137 (9)	0.0234 (4)	
C15	0.8584 (2)	−0.3463 (3)	0.34161 (9)	0.0216 (4)	
H15A	0.8551	−0.4936	0.3405	0.026*	
H15B	0.9586	−0.3055	0.3654	0.026*	
C16	0.8401 (2)	−0.2664 (3)	0.27157 (9)	0.0211 (4)	
H16A	0.9197	−0.3248	0.2488	0.025*	
H16B	0.7402	−0.3082	0.2478	0.025*	
C17	0.8514 (2)	−0.0399 (3)	0.26976 (9)	0.0217 (4)	
H17A	0.7597	0.0183	0.2841	0.026*	
H17B	0.9405	0.0033	0.3011	0.026*	
C18	0.9069 (2)	0.2494 (3)	0.20824 (8)	0.0176 (3)	
H18A	1.0014	0.2662	0.2398	0.021*	
H18B	0.8254	0.3252	0.2248	0.021*	
C19	0.93024 (18)	0.3315 (3)	0.14215 (8)	0.0165 (3)	
H19A	0.9583	0.4740	0.1466	0.020*	
H19B	1.0143	0.2593	0.1262	0.020*	
C20	0.7427 (2)	0.0956 (3)	0.09013 (9)	0.0204 (4)	
H20A	0.8210	0.0165	0.0724	0.025*	
H20B	0.6459	0.0810	0.0599	0.025*	
C21	0.72335 (19)	0.0166 (3)	0.15757 (9)	0.0190 (4)	
H21A	0.6415	0.0913	0.1744	0.023*	
H21B	0.6936	−0.1254	0.1540	0.023*	
C22	0.8046 (2)	0.3855 (3)	0.02724 (9)	0.0215 (4)	
H22A	0.7107	0.3553	−0.0034	0.026*	
H22B	0.8897	0.3155	0.0113	0.026*	
C23	0.8335 (3)	0.6079 (4)	0.02694 (10)	0.0303 (5)	
H23A	0.7507	0.6790	0.0443	0.036*	
H23B	0.9302	0.6388	0.0555	0.036*	
C24	0.2136 (2)	0.9043 (3)	0.17266 (9)	0.0206 (4)	
C25	0.3389 (2)	0.7804 (3)	0.15514 (10)	0.0253 (4)	
H25A	0.4392	0.8292	0.1675	0.030*	
C26	0.3240 (2)	0.6105 (3)	0.12434 (9)	0.0221 (4)	
H26A	0.2244	0.5607	0.1108	0.027*	
C27	0.4562 (2)	0.4892 (3)	0.10914 (9)	0.0222 (4)	

### Atomic displacement parameters (Å^2^)

**Table:**

	*U*^11^	*U*^22^	*U*^33^	*U*^12^	*U*^13^	*U*^23^
O1	0.0248 (7)	0.0447 (10)	0.0304 (8)	0.0030 (7)	0.0062 (6)	0.0185 (7)
O2	0.0169 (6)	0.0203 (7)	0.0326 (7)	0.0022 (6)	0.0074 (5)	−0.0049 (6)
O3	0.0236 (7)	0.0249 (9)	0.0471 (9)	0.0008 (6)	0.0016 (6)	−0.0126 (7)
O4	0.0235 (7)	0.0433 (11)	0.0557 (10)	0.0013 (7)	0.0033 (7)	−0.0287 (9)
O5	0.0152 (6)	0.0247 (8)	0.0363 (7)	0.0016 (6)	0.0042 (5)	−0.0090 (7)
N1	0.0218 (7)	0.0212 (8)	0.0198 (7)	0.0012 (7)	0.0082 (6)	−0.0006 (7)
N2	0.0163 (6)	0.0136 (7)	0.0175 (7)	0.0013 (6)	0.0043 (5)	0.0000 (6)
N3	0.0172 (6)	0.0139 (7)	0.0173 (7)	0.0022 (6)	0.0028 (5)	−0.0014 (6)
C1	0.0241 (8)	0.0221 (10)	0.0206 (8)	−0.0026 (8)	0.0079 (7)	0.0007 (8)
C2	0.0331 (10)	0.0281 (11)	0.0271 (9)	0.0060 (10)	0.0090 (8)	0.0022 (9)
C3	0.0357 (11)	0.0435 (15)	0.0262 (10)	0.0039 (11)	0.0038 (9)	0.0091 (10)
C4	0.0356 (11)	0.0482 (16)	0.0206 (9)	−0.0090 (11)	0.0060 (8)	−0.0023 (10)
C5	0.0380 (11)	0.0342 (13)	0.0306 (10)	−0.0051 (10)	0.0158 (9)	−0.0092 (10)
C6	0.0298 (9)	0.0217 (10)	0.0269 (9)	0.0001 (8)	0.0101 (7)	−0.0026 (8)
C7	0.0455 (13)	0.0267 (12)	0.0393 (12)	0.0123 (11)	0.0165 (10)	−0.0011 (10)
C8	0.0395 (12)	0.0334 (14)	0.0406 (12)	0.0163 (11)	0.0138 (10)	0.0101 (11)
C9	0.0280 (9)	0.0367 (13)	0.0247 (9)	0.0050 (9)	0.0074 (7)	0.0121 (9)
C10	0.0260 (10)	0.0648 (19)	0.0329 (11)	0.0034 (12)	0.0044 (9)	0.0224 (13)
C11	0.0315 (11)	0.071 (2)	0.0311 (11)	−0.0149 (13)	−0.0037 (9)	0.0218 (13)
C12	0.0520 (14)	0.0496 (16)	0.0240 (10)	−0.0233 (13)	0.0004 (9)	0.0082 (11)
C13	0.0379 (11)	0.0331 (13)	0.0235 (9)	−0.0075 (10)	0.0057 (8)	0.0036 (9)
C14	0.0247 (9)	0.0285 (11)	0.0177 (8)	−0.0023 (8)	0.0054 (7)	0.0067 (8)
C15	0.0249 (9)	0.0187 (9)	0.0226 (9)	0.0044 (8)	0.0082 (7)	−0.0004 (8)
C16	0.0275 (9)	0.0163 (9)	0.0214 (8)	0.0010 (8)	0.0096 (7)	−0.0018 (8)
C17	0.0301 (9)	0.0190 (9)	0.0176 (8)	0.0003 (8)	0.0086 (7)	−0.0002 (7)
C18	0.0223 (8)	0.0127 (9)	0.0180 (8)	−0.0017 (7)	0.0032 (6)	−0.0019 (7)
C19	0.0145 (7)	0.0156 (9)	0.0193 (8)	−0.0013 (7)	0.0019 (6)	−0.0004 (7)
C20	0.0234 (8)	0.0139 (9)	0.0228 (9)	−0.0006 (7)	−0.0006 (7)	−0.0024 (8)
C21	0.0159 (7)	0.0158 (9)	0.0254 (9)	−0.0017 (7)	0.0039 (6)	0.0000 (7)
C22	0.0260 (9)	0.0226 (10)	0.0162 (8)	0.0033 (8)	0.0043 (7)	0.0005 (7)
C23	0.0420 (12)	0.0236 (11)	0.0250 (10)	−0.0059 (10)	0.0040 (8)	0.0058 (9)
C24	0.0177 (8)	0.0202 (10)	0.0239 (9)	0.0009 (7)	0.0029 (7)	−0.0018 (8)
C25	0.0158 (8)	0.0266 (11)	0.0341 (10)	0.0008 (8)	0.0063 (7)	−0.0017 (9)
C26	0.0154 (8)	0.0257 (10)	0.0254 (9)	0.0035 (7)	0.0031 (7)	−0.0008 (8)
C27	0.0190 (8)	0.0263 (11)	0.0221 (9)	0.0028 (8)	0.0056 (7)	−0.0014 (8)

### Geometric parameters (Å, °)

**Table:**

O1—C23	1.404 (2)	C10—H10A	0.9500
O1—H1O	0.848 (17)	C11—C12	1.374 (4)
O2—C24	1.322 (2)	C11—H11A	0.9500
O2—H2O	0.866 (16)	C12—C13	1.394 (3)
O3—C24	1.216 (3)	C12—H12A	0.9500
O4—C27	1.243 (3)	C13—C14	1.393 (3)
O5—C27	1.262 (2)	C13—H13A	0.9500
N1—C1	1.427 (2)	C15—C16	1.527 (3)
N1—C14	1.432 (2)	C15—H15A	0.9900
N1—C15	1.463 (2)	C15—H15B	0.9900
N2—C18	1.469 (2)	C16—C17	1.525 (3)
N2—C21	1.473 (2)	C16—H16A	0.9900
N2—C17	1.485 (2)	C16—H16B	0.9900
N3—C20	1.491 (3)	C17—H17A	0.9900
N3—C19	1.493 (2)	C17—H17B	0.9900
N3—C22	1.495 (2)	C18—C19	1.514 (2)
N3—H3N	0.900 (16)	C18—H18A	0.9900
C1—C2	1.386 (3)	C18—H18B	0.9900
C1—C6	1.411 (3)	C19—H19A	0.9900
C2—C3	1.392 (3)	C19—H19B	0.9900
C2—H2A	0.9500	C20—C21	1.523 (3)
C3—C4	1.376 (4)	C20—H20A	0.9900
C3—H3A	0.9500	C20—H20B	0.9900
C4—C5	1.373 (4)	C21—H21A	0.9900
C4—H4A	0.9500	C21—H21B	0.9900
C5—C6	1.400 (3)	C22—C23	1.516 (3)
C5—H5A	0.9500	C22—H22A	0.9900
C6—C7	1.459 (3)	C22—H22B	0.9900
C7—C8	1.330 (4)	C23—H23A	0.9900
C7—H7A	0.9500	C23—H23B	0.9900
C8—C9	1.461 (4)	C24—C25	1.480 (3)
C8—H8A	0.9500	C25—C26	1.304 (3)
C9—C14	1.403 (3)	C25—H25A	0.9500
C9—C10	1.406 (3)	C26—C27	1.504 (3)
C10—C11	1.374 (5)	C26—H26A	0.9500
			
C23—O1—H1O	105.4 (19)	H15A—C15—H15B	108.0
C24—O2—H2O	109.7 (17)	C17—C16—C15	112.06 (17)
C1—N1—C14	114.32 (14)	C17—C16—H16A	109.2
C1—N1—C15	116.87 (15)	C15—C16—H16A	109.2
C14—N1—C15	117.15 (16)	C17—C16—H16B	109.2
C18—N2—C21	108.45 (14)	C15—C16—H16B	109.2
C18—N2—C17	109.56 (14)	H16A—C16—H16B	107.9
C21—N2—C17	111.93 (14)	N2—C17—C16	112.78 (16)
C20—N3—C19	109.17 (14)	N2—C17—H17A	109.0
C20—N3—C22	110.20 (15)	C16—C17—H17A	109.0
C19—N3—C22	112.96 (14)	N2—C17—H17B	109.0
C20—N3—H3N	112.8 (15)	C16—C17—H17B	109.0
C19—N3—H3N	106.1 (14)	H17A—C17—H17B	107.8
C22—N3—H3N	105.6 (14)	N2—C18—C19	111.15 (15)
C2—C1—C6	119.75 (18)	N2—C18—H18A	109.4
C2—C1—N1	121.72 (18)	C19—C18—H18A	109.4
C6—C1—N1	118.46 (18)	N2—C18—H18B	109.4
C1—C2—C3	120.5 (2)	C19—C18—H18B	109.4
C1—C2—H2A	119.8	H18A—C18—H18B	108.0
C3—C2—H2A	119.8	N3—C19—C18	110.28 (14)
C4—C3—C2	120.1 (2)	N3—C19—H19A	109.6
C4—C3—H3A	120.0	C18—C19—H19A	109.6
C2—C3—H3A	120.0	N3—C19—H19B	109.6
C5—C4—C3	119.94 (19)	C18—C19—H19B	109.6
C5—C4—H4A	120.0	H19A—C19—H19B	108.1
C3—C4—H4A	120.0	N3—C20—C21	110.82 (15)
C4—C5—C6	121.6 (2)	N3—C20—H20A	109.5
C4—C5—H5A	119.2	C21—C20—H20A	109.5
C6—C5—H5A	119.2	N3—C20—H20B	109.5
C5—C6—C1	118.1 (2)	C21—C20—H20B	109.5
C5—C6—C7	119.5 (2)	H20A—C20—H20B	108.1
C1—C6—C7	122.37 (18)	N2—C21—C20	110.51 (14)
C8—C7—C6	127.4 (2)	N2—C21—H21A	109.5
C8—C7—H7A	116.3	C20—C21—H21A	109.5
C6—C7—H7A	116.3	N2—C21—H21B	109.5
C7—C8—C9	126.4 (2)	C20—C21—H21B	109.5
C7—C8—H8A	116.8	H21A—C21—H21B	108.1
C9—C8—H8A	116.8	N3—C22—C23	112.42 (17)
C14—C9—C10	118.1 (2)	N3—C22—H22A	109.1
C14—C9—C8	122.6 (2)	C23—C22—H22A	109.1
C10—C9—C8	119.3 (2)	N3—C22—H22B	109.1
C11—C10—C9	121.4 (3)	C23—C22—H22B	109.1
C11—C10—H10A	119.3	H22A—C22—H22B	107.9
C9—C10—H10A	119.3	O1—C23—C22	109.73 (19)
C10—C11—C12	120.3 (2)	O1—C23—H23A	109.7
C10—C11—H11A	119.9	C22—C23—H23A	109.7
C12—C11—H11A	119.9	O1—C23—H23B	109.7
C11—C12—C13	119.9 (3)	C22—C23—H23B	109.7
C11—C12—H12A	120.1	H23A—C23—H23B	108.2
C13—C12—H12A	120.1	O3—C24—O2	123.43 (17)
C14—C13—C12	120.4 (2)	O3—C24—C25	121.94 (17)
C14—C13—H13A	119.8	O2—C24—C25	114.62 (17)
C12—C13—H13A	119.8	C26—C25—C24	125.90 (18)
C13—C14—C9	119.92 (19)	C26—C25—H25A	117.0
C13—C14—N1	121.6 (2)	C24—C25—H25A	117.0
C9—C14—N1	118.5 (2)	C25—C26—C27	123.45 (17)
N1—C15—C16	111.39 (16)	C25—C26—H26A	118.3
N1—C15—H15A	109.4	C27—C26—H26A	118.3
C16—C15—H15A	109.4	O4—C27—O5	124.04 (18)
N1—C15—H15B	109.4	O4—C27—C26	118.37 (17)
C16—C15—H15B	109.4	O5—C27—C26	117.59 (18)
			
C14—N1—C1—C2	113.7 (2)	C8—C9—C14—N1	−6.2 (3)
C15—N1—C1—C2	−28.5 (3)	C1—N1—C14—C13	−111.4 (2)
C14—N1—C1—C6	−69.6 (2)	C15—N1—C14—C13	30.7 (3)
C15—N1—C1—C6	148.23 (18)	C1—N1—C14—C9	71.6 (2)
C6—C1—C2—C3	−1.5 (3)	C15—N1—C14—C9	−146.34 (18)
N1—C1—C2—C3	175.2 (2)	C1—N1—C15—C16	−156.00 (18)
C1—C2—C3—C4	1.4 (3)	C14—N1—C15—C16	62.9 (2)
C2—C3—C4—C5	−0.8 (4)	N1—C15—C16—C17	61.6 (2)
C3—C4—C5—C6	0.2 (3)	C18—N2—C17—C16	−168.59 (16)
C4—C5—C6—C1	−0.2 (3)	C21—N2—C17—C16	71.1 (2)
C4—C5—C6—C7	−179.9 (2)	C15—C16—C17—N2	168.13 (14)
C2—C1—C6—C5	0.8 (3)	C21—N2—C18—C19	−60.33 (17)
N1—C1—C6—C5	−175.95 (18)	C17—N2—C18—C19	177.23 (14)
C2—C1—C6—C7	−179.5 (2)	C20—N3—C19—C18	−56.34 (18)
N1—C1—C6—C7	3.7 (3)	C22—N3—C19—C18	−179.32 (16)
C5—C6—C7—C8	−147.0 (2)	N2—C18—C19—N3	59.58 (19)
C1—C6—C7—C8	33.4 (4)	C19—N3—C20—C21	56.30 (18)
C6—C7—C8—C9	0.8 (4)	C22—N3—C20—C21	−179.09 (14)
C7—C8—C9—C14	−33.1 (3)	C18—N2—C21—C20	59.56 (18)
C7—C8—C9—C10	145.5 (2)	C17—N2—C21—C20	−179.45 (16)
C14—C9—C10—C11	0.7 (3)	N3—C20—C21—N2	−58.89 (19)
C8—C9—C10—C11	−177.9 (2)	C20—N3—C22—C23	173.51 (16)
C9—C10—C11—C12	0.7 (3)	C19—N3—C22—C23	−64.1 (2)
C10—C11—C12—C13	−0.9 (3)	N3—C22—C23—O1	−177.46 (15)
C11—C12—C13—C14	−0.4 (3)	O3—C24—C25—C26	−176.8 (2)
C12—C13—C14—C9	1.8 (3)	O2—C24—C25—C26	4.6 (3)
C12—C13—C14—N1	−175.25 (18)	C24—C25—C26—C27	−178.66 (18)
C10—C9—C14—C13	−1.9 (3)	C25—C26—C27—O4	−170.4 (2)
C8—C9—C14—C13	176.66 (19)	C25—C26—C27—O5	9.6 (3)
C10—C9—C14—N1	175.19 (18)		

### Hydrogen-bond geometry (Å, °)

**Table:**

Cg2 and Cg3 are the centroids of the C1–C6 and C9–C14 rings, respectively.

**Table:**

*D*—H···*A*	*D*—H	H···*A*	*D*···*A*	*D*—H···*A*
O1—H1O···O4^i^	0.85 (2)	1.83 (2)	2.674 (2)	171 (3)
O2—H2O···N2^ii^	0.87 (2)	1.79 (2)	2.649 (2)	176 (3)
N3—H3N···O5	0.90 (2)	1.72 (2)	2.616 (2)	179 (2)
N3—H3N···O4	0.90 (2)	2.59 (2)	3.167 (2)	123.(2)
C12—H12A···O3^iii^	0.95	2.49	3.399 (3)	159.
C19—H19A···O2^iv^	0.99	2.57	3.551 (2)	170.
C19—H19B···O1^v^	0.99	2.47	3.365 (2)	151.
C21—H21B···O5^vi^	0.99	2.43	3.415 (2)	172.
C22—H22B···O1^v^	0.99	2.57	3.447 (2)	148.
C2—H2A···Cg2^vii^	0.95	2.95	3.684 (2)	135
C5—H5A···Cg3^viii^	0.95	2.79	3.655 (2)	152

Symmetry codes: (i) −*x*+1, *y*+1/2, −*z*; (ii) *x*−1, *y*+1, *z*; (iii) *x*, *y*−2, *z*; (iv) *x*+1, *y*, *z*; (v) −*x*+2, *y*−1/2, −*z*; (vi) *x*, *y*−1, *z*; (vii) −*x*+2, *y*−1/2, −*z*+1; (viii) −*x*+1, *y*+1/2, −*z*+1.
